# The correlation between the first heart sound and cardiac output as measured by using digital esophageal stethoscope under anaesthesia

**Published:** 2014

**Authors:** Young Duck Shin, Kyoung Hoon Yim, Sang Hi Park, Yong Wook Jeon, Jin Ho Bae, Tae Soo Lee, Myoung Hwan Kim, Young Jin Choi

**Affiliations:** 1 Young Duck Shin, Department of Anesthesiology and Pain Medicine, Chungbuk National University Hospital, Korea.; 2 Kyoung Hoon Yim, Department of Anesthesiology and Pain Medicine, Chungbuk National University Hospital, Korea.; 3 Sang Hi Park, Department of Anesthesiology and Pain Medicine, Chungbuk National University Hospital, Korea.; 4 Yong Wook Jeon, Department of Anesthesiology and Pain Medicine, Chungbuk National University Hospital, Korea.; 5 Jin Ho Bae, Dept. of Anesthesiology and Pain Medicine, College of Medicine, Chungbuk National University, Korea.; 6 Tae Soo Lee, Dept. of Biomedical Engineering, College of Medicine, Chungbuk National University, Korea.; 7 Myoung Hwan Kim, Laboratory Animal Research Center, Chungbuk National University, Korea.; 8 Young Jin Choi, Department of Surgery, College of Medicine, Eulji University, Korea.

**Keywords:** Cardiac output, First heart sound, Heart rate, Stethoscope

## Abstract

***Objective:*** The use of an esophageal stethoscope is a basic heart sounds monitoring procedure performed in patients under general anesthesia. As the size of the first heart sound can express the left ventricle function, its correlation with cardiac output should be investigated. The aim of this study was to investigate the effects of cardiac output (CO) on the first heart sound (S1) amplitude.

***Methods*** *:* Six male beagles were chosen. The S1 was obtained with the newly developed esophageal stethoscope system. CO was measured using NICOM, a non-invasive CO measuring device. Ephedrine and beta blockers were administered to the subjects to compare changes in figures, and the change from using an inhalation anesthetic was also compared.

***Results:*** The S1 amplitude displayed positive correlation with the change rate of CO (r = 0.935, *p *< 0.001). The heart rate measured using the esophageal stethoscope and ECG showed considerably close figures through the Bland-Altman plot and showed a high positive correlation (r = 0.988, p < 0,001).

***Conclusion:*** In beagles, the amplitude of S1 had a significant correlation with changes in CO in a variety of situations.

## INTRODUCTION

Listening to heart sounds through a stethoscope is one of the basic examinations^1^ and should be done in operating rooms. Listening to heart sounds is also used in monitoring for arrhythmia and diagnosing heart disease.^2^^-^^4^ However, traditional chest auscultation can be impossible depending on surgery, and extraneous noise can occur due to body movement. Its usefulness also depends heavily on body weight and air movement in the thoracic cage. On the other hand, the esophageal stethoscope is advantageous in continuous evaluation of heart sounds because it can be placed in close proximity to the heart and undergoes minimal changes once it is fixed. The esophageal stethoscope can measure central temperature and has even been studied in the measurement of the residual flow of patients with patent ductus arteriosus.^5^ However, the use of the esophageal stethoscope has decreased with the development of transesophageal ultrasound. One of the reasons for the decline in use is that the obtained heart sound is not expressed objectively but instead relies on the judgment of the doctor and, thus, could be interpreted differently according to the expertise of the individual.

This study visualized heart sounds using the esophageal stethoscope and analyzed them quantitatively to investigate whether they can be used to evaluate heart function during surgery. To accomplish this, a new program and mechanical equipment were needed in addition to the existing esophageal stethoscope. Newly developed technology was used for the mechanical equipment and program (digital esophageal stethoscope system, DESS, Park SH et al.^6^, Korea). This system digitizes the analogue heart sound input for display and uses a program to perform noise removal and amplification. Our Laboratory has studied heart sounds.^6^ Based on previous study (Park et al.^6^, Heart sounds analysis via esophageal stethoscope system in beagles, 2013, (DOI) 10.1007/s10877-013-9459-0), this study was conducted.

Heart sounds comprise two main components: the first heart sound (S1), and the second heart sound (S2).^7^ This research analyzed the first heart sound, which expresses the function of the left ventricle. The amplitude of S1 has a correlation with the contractile force of the left ventricle and an increase in heart rate (HR). The hypothesis of this research was that a correlation exists between S1 amplitude with stroke volume (SV) and cardiac output (CO).

There are numerous methods to measure cardiac output continuously and in real time. However, in current clinical practice, non-invasive tools are being developed for use. Typical methods include the esophageal Doppler method,^8^^,^^9^ the thoracic bioimpedance method,^10^ and the bioreactance method.^11^^,^^12^ Instead of the traditional thermal dilution method, this research used the bioreactance equipment NICOM (Cheetah Medical, Portland, OR), which is used in current clinical practice for monitoring heart sounds and analyzing correlations with S1, as it is non-invasive and has superior accuracy. In addition, to investigate whether the esophageal stethoscope can function as an independent monitor, the heart rate was obtained through S1 and compared with ECG.

The hypothesis of this research was that the change in S1 amplitude obtained through the esophageal stethoscope has a correlation with cardiac output. An animal experiment was conducted to prove this hypothesis.

## METHODS

**Fig.1:**
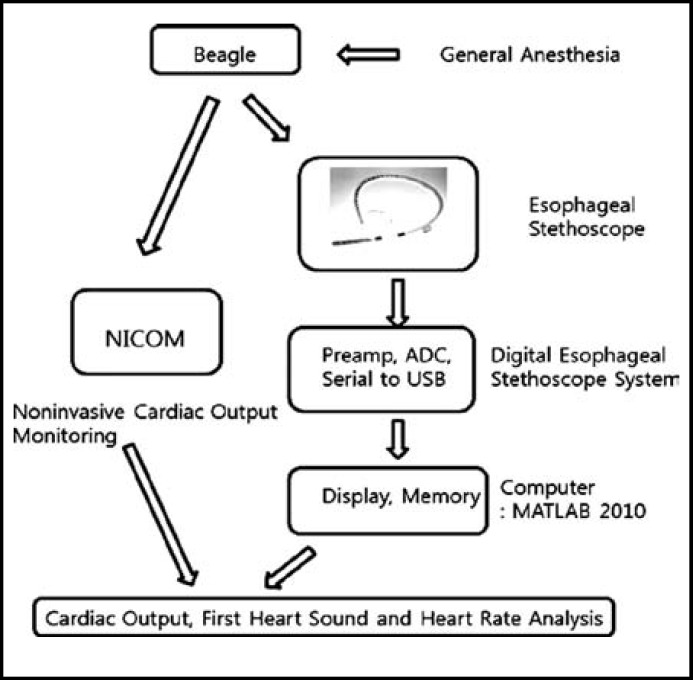
Diagram of Experimental Process

**Fig.2:**
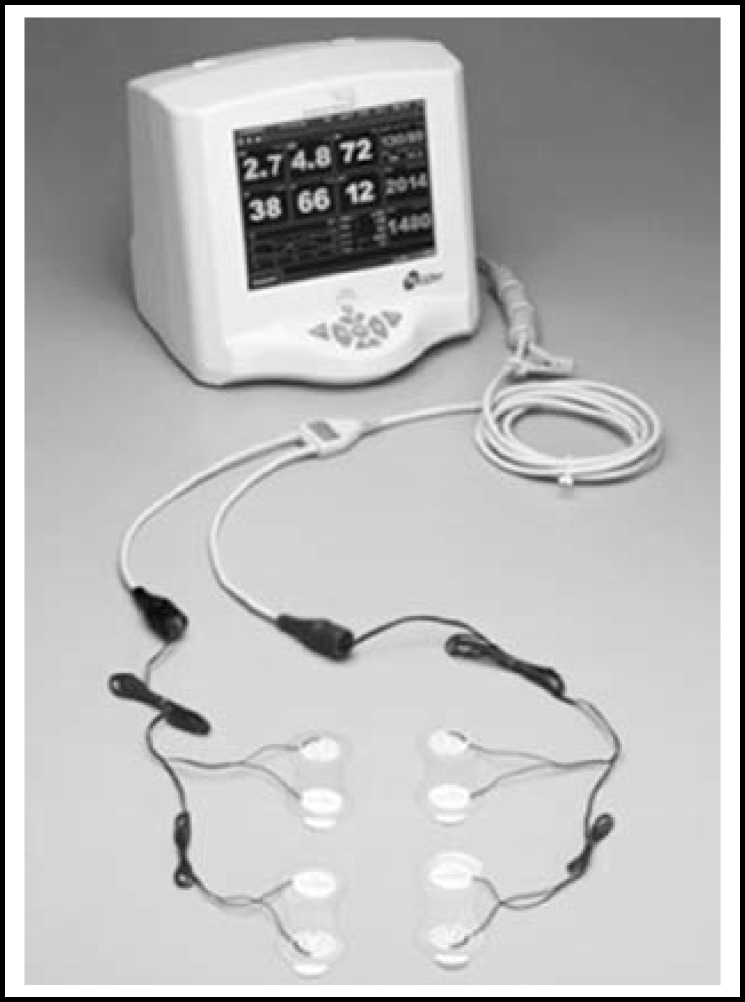
NICOM noninvasive Cardiac Output Monitor. (Copyright www.cheetah-medical.com

Breeding and experimenting with experimental animals followed the regulations set by the animal experiment ethics committee of the Chungbuk National University Experimental Animal Research Support Center (CBNUA-564-13-01). Six male beagles from 10 to 12 kg (11.1 ± 0.63) were chosen. Atropine sulfate 0.02 mg/kg was administered for premedication, and anesthesia was induced by subcutaneous injection of 2% xylazine hydrochloride 1 mg/kg. Isoflurane was used to induce inhalation anesthesia, and endotracheal intubation was performed through the oral cavity. Intermittent positive pressure ventilation was performed for respiration. The subjects were stabilized in a left lying position, and the esophageal stethoscope (Deoyal®, USA) was fixed at the position where the S1 sound was heard the loudest. The microphone, part of the digital heart sound measuring equipment, was connected to the esophageal stethoscope. The arterial pressure was measured via femoral artery. After the vital signs were stabilized, the S1 amplitude and standard for cardiac output were measured. The digital esophageal stethoscope system hardware comprises a microphone, microcontroller, amplifier, digital converter, and power connecting equipment. The heart sound analysis program recorded the data transmitted through the USB, while simultaneously visualizing the data. The MATLAB program (version R2009z, MathWorks Inc., USA) was used for the Heart Sound Analyser 1.0 developed by Park SH et al.^6^ This quantifies and expresses the amplitude of the heart sound and shows the cardiac cycle and heart rate (Fig.1). The heart sound data were stored in the wav file type, and the stored wav files were loaded for replay and analysis. The S1 amplitude was calculated as the mean value of data for three seconds. The heart rate was calculated using the heart cycle of the S1 amplitude, and results were compared to those measured using the ECG. The non-invasive cardiac output measuring device NICOM (Cheetah Medical, Portland, OR) was used in the experiment to measure cardiac output (Fig.2). 

**Fig.3:**
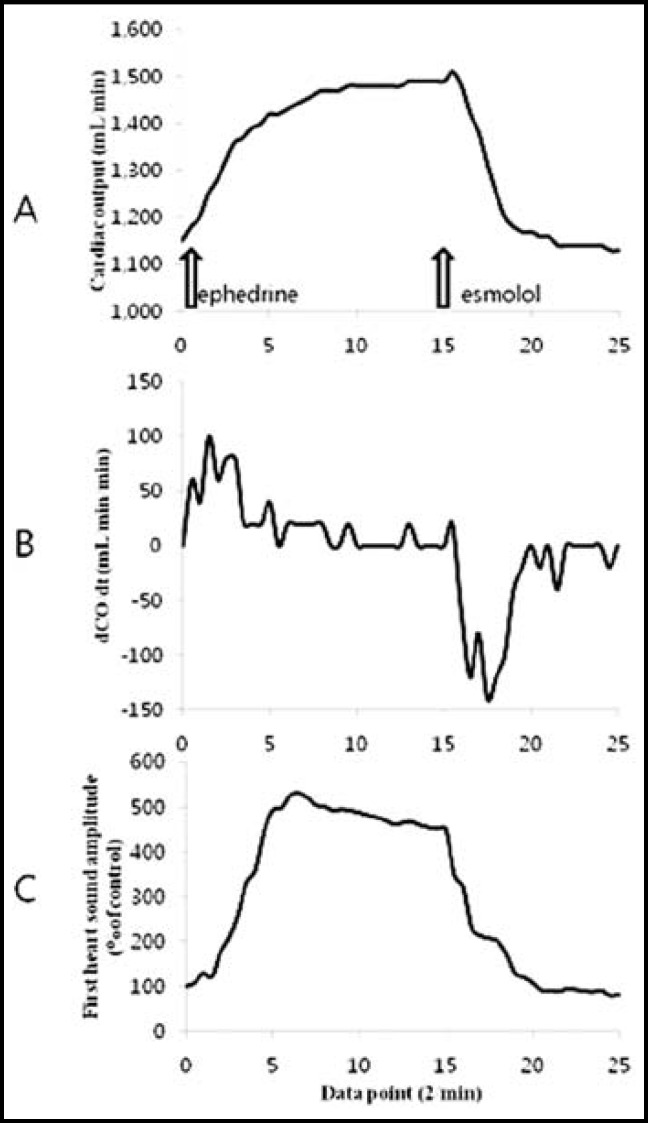
Cardiac output monitoring test. Panel A. Cardiac output during variation in cardiac inotropy and β-blockade. Panel B. The derivative cardiac output (dCO/dt), with peak positive and negative deflections representing the maximal rate of change during events depicted in panel A. Panel C. First heart sound during variation in cardiac inotropy and β-blockade

**Fig.4:**
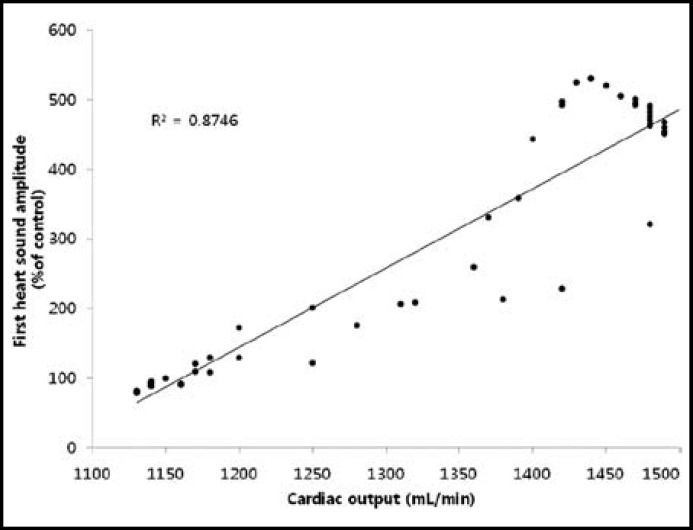
Relationship of first heart sound amplitude to the cardiac output obtained in 6 animals during 2 experimental procedures and 50 cardiac cycles. The values have a correlation coefficient of 0.935 (*p*<0.001).

**Fig.5:**
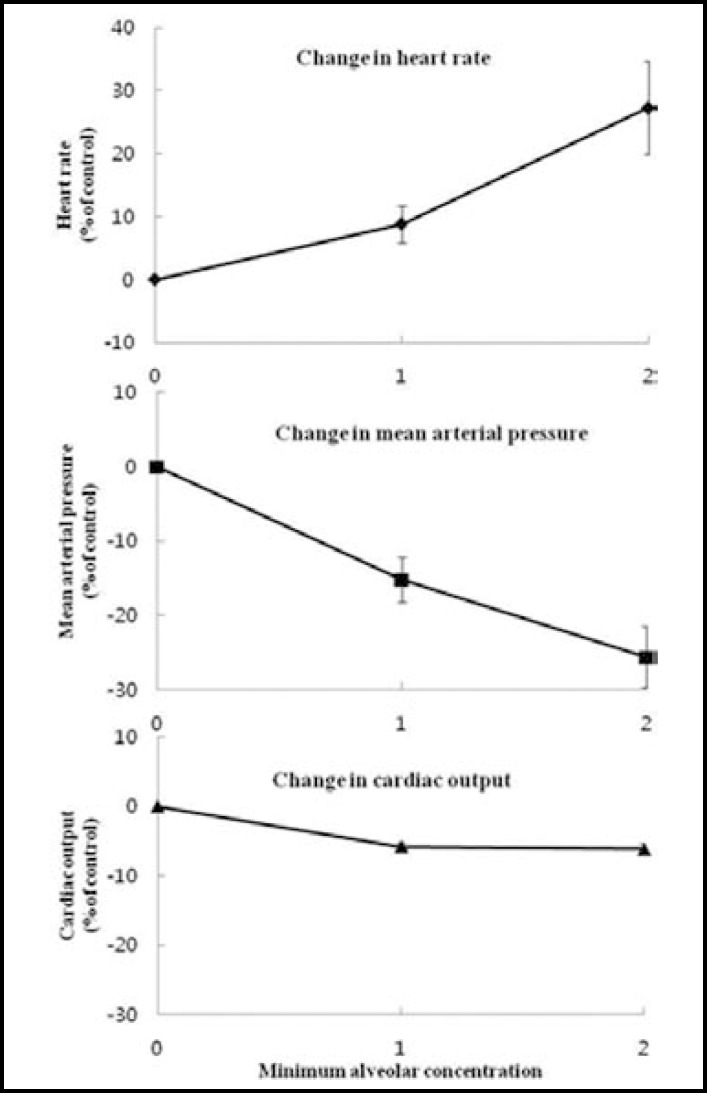
Heart rate, mean arterial pressure, and cardiac output changes in animals receiving general anesthesia with isoflurane. Isoflurane caused increase in heart rate. Decrease in mean arterial pressure from isoflurane might be due to venodilation. Isoflurane produced little change in cardiac output

**Fig.6:**
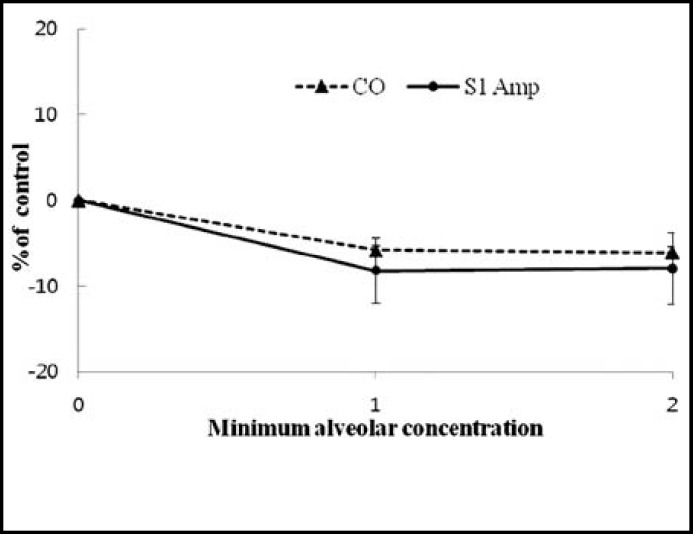
Cardiac output and first heart sound amplitude little change in isoflurane minimum alveolar concentration. There were no significant differences

**Fig.7:**
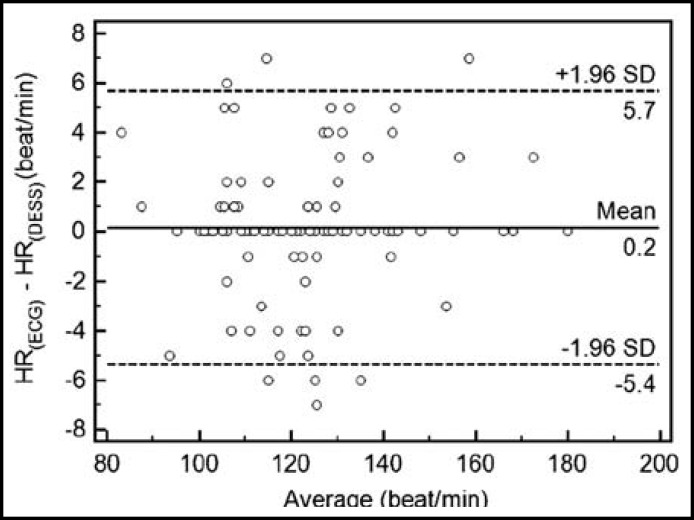
Bland-Altman plot shows correlation between ECG and DESS. There was significant correlation between methods (r=0.988, p<0.001). DESS: Digital Esophageal Stethoscope System

Situations similar to actual clinical scenarios were designed to investigate the correlation between S1 amplitude and cardiac output when inotropic agents and beta blockers were used. In these conditions, it was hypothesized that cardiac output and arterial pressure would show an accompanying increase and decrease. Ephedrine (0.2 mg/kg) was used as the inotropic agent and esmolol (1 mg/kg) as the beta blocker. Second, the change in S1 amplitude was examined when maintaining cardiac output while decreasing arterial pressure using isoflurane. The standard value was measured, and the Minimum Alveolar Concentration (MAC) of isoflurane was changed from 1MAC to 2MAC. Each variable—cardiac output, heart sound, and arterial pressure—was checked at 30-second intervals after measuring the standard value, and they were measured in end-expiratory pressure to reduce the interference of respiration sound. To compare values, heart rate was measured simultaneously with the esophageal stethoscope and ECG 100 times each using the pressor agent and beta blocker. 

SPSS (version 12.0, SPSS Inc, Chicago, IL, USA) was used for statistical analysis. The changes in cardiac output, arterial pressure, and S1 amplitude were analyzed with the Pearson’s correlation coefficient method. The changes in S1 amplitude, cardiac output, heart rate, and arterial pressure with increasing concentrations of the inhalation anesthetic were compared with the standard value using one-way analysis of variance. A Bland-Altman plot was made using the MedCalc (version 12.3.0, MedCalc Software, Mariakerke, Belgium) program to compare heart rate in the esophageal stethoscope and ECG, and the correlation between the two measurements was analyzed using the Pearson’s correlation coefficient method. Statistical significance was defined as having a p-value of less than 0.05.

## RESULTS

The change rate in cardiac output and S1 amplitude changed according to the administration of the inotropic agent and beta blocker. The change in cardiac output per unit time (dCO/dt) showed a direct response after administering the pressor agent and beta blocker, and the graph showed differences with the change rate of S1 amplitude (Fig.3). The change in cardiac output and S1 amplitude were measured a total of 50 times, and there was a strong positive correlation (r = 0.935, p < 0.001) (Fig.4).

When the concentration of isoflurane was increased to 2MAC, the mean arterial pressure decreased (25.6% ± 4.12%), while heart rate significantly increased (27.3% ± 7.4%) (Fig.5). However, the change in S1 amplitude and cardiac output did not show a significant change according to concentration compared to the standard value in both 1MAC and 2MAC (Fig.6).

The heart rate from the esophageal stethoscope and ECG was expressed through a Bland-Altman plot (Fig.7). Within a 97% confidence interval, about 93% were included. This shows that the heart rate obtained from the esophageal stethoscope and ECG were considerably close, and the correlation analysis also showed a high positive correlation (r = 0.988, p < 0.001).

## DISCUSSION

It is important to note that heart sounds have waveforms. This research calculated the root mean square (RMS) of the sound waveform to apply the size of the changing value. There has been precedent study reporting a positive correlation between S1 amplitude with SV and CO (r = 0.76).^13^ In these studies, however, heart sound amplitude was calculated using only the amplitude height; this is not considered to be very accurate, as the sound characteristics were not reflected fully. Our research calculated the RMS of the sound waveform to apply the size of the changing value. 

The first heart sound is made by the closing of atrioventricular valves and is heard loudly in the apex; the second heart sound is made by the closing of the aortic valve and pulmonary valve and is heard loudly in the base of the heart.^14^ This experiment analyzed S1, but much research is being conducted on S2.^15^^,^^16^

To the present, there has been multidimensional research on heart sound, which has been analyzed as a major factor in many studies regarding its relationship with respiration,^17^ estimating pulmonary arterial pressure,^18^ detecting heart disease,^4^ and evaluating left ventricle function.^19^ S1 is related to the maximum speed of increase in left ventricle systolic pressure (maximum dP/dt). This is because the isovolumic contraction period of the left ventricle is when the first heart sound is made, and as the pressure increases, the sound becomes larger accordingly.

In this experiment, the first heart sound showed a high correlation with changes in cardiac output. The contractile power of the left ventricle would have increased when inotropic agents were used, and this would make the closing of the valves stronger. The opposite situation would have arisen when using beta blockers. In addition, the S1 amplitude was maintained when cardiac output was maintained using isoflurane. Isoflurane is the only inhalation anesthetic that maintains rather than decreases cardiac output when the concentration is increased.^20^

The fact that the first heart sound has a correlation with cardiac output is very meaningful in various situations, and it is more significant given the fact that the heart rate can be deduced independently. It is highly probable that surrounding noise that was not completely removed accounted for the slight difference in heart rate between the esophageal stethoscope and ECG. Although not separated and processed statistically, almost exact concordance was shown in a quiet situation of end-expiratory period during the experiment.

Continuous monitoring of cardiac output is very important during surgery or when treating critical patients. The importance increases when changes become extreme due to unstable hemodynamics. Monitoring will be meaningful if the S1 amplitude, which is proportionate to left ventricle contractile force, shows a correlation with cardiac output. Recently, there has been active development of non-invasive tools to measure cardiac output in clinical practice. These are being used in many studies as indicators of hydration as well as for the evaluation of heart function.

There are limitations to applying this experiment directly to clinical situations. An important point is whether the correlation found in animals can be similarly applied to adult humans. In this experiment, subjects were limited to dogs ranging in weight from 10 to 12 kg, which was the experimental model in Heerdt et al.^21^ In that study, when the NICOM device set the age of beagles weighing 12 kg or less at 7 years, it showed accurate concordance with cardiac output measured through the aorta in an invasive method. Of course, the relationship between adult heart function and heart sound obtained through precordial auscultation has been researched before, but almost no research has been conducted regarding the esophageal stethoscope. In addition, this experiment was conducted on normal dogs with no problems in their heart or valves in a heart ultrasound performed before the experiment. There are reports that heart sound differs before and after heart surgery.^22^^,^^23^ The largest factor involved in heart sounds is the closing of valves. If the mitral valve cannot close properly due to disease or calcification, the first heart sound can be small or difficult to detect. If research is conducted on hearts with disease, further relationships may be revealed.

The heart sound amplitudes in the subjects were expressed through ratios to the standard value. Generally, the amplitude of sound is expressed by decibel (dB), and the smallest sound that can be heard by normal adult ears is 0 dB. If the normal amplitude of heart sounds is expressed as it is, it will be minus dB. Therefore, this experiment calculated the change rate with heart sounds in a stable state as the standard value.

## CONCLUSION

In conclusion, the esophageal stethoscope is a non-invasive tool that can be placed in close proximity to the heart for monitoring. Through this experiment, it was revealed that the amplitude of the first heart sound has a correlation with cardiac output. In addition, the heart rate was accurately measured through the first heart sound. These results are considered to be meaningful for the goal to accurately monitor cardiac output safely and economically.

## Authors Contribution:

Young Duck Shin & Kyoung Hoon Yim: Conceived, Designed and did statistical analysis & editing of manuscript

Sang Hi Park, Yong Wook Jeon, Jin Ho Bae, Tae Soo Lee, Myoung Hwan Kim & Young Jin Choi: Did data collection and manuscript writing.

Young Duck Shin: Did review and final approval of manuscript.
